# The Collaborative Assessment and Management of Suicidality compared to enhanced treatment as usual for inpatients who are suicidal: A randomized controlled trial

**DOI:** 10.3389/fpsyt.2023.1038302

**Published:** 2023-03-02

**Authors:** Miriam Santel, Frank Neuner, Michaela Berg, Carolin Steuwe, David A. Jobes, Martin Driessen, Thomas Beblo

**Affiliations:** ^1^Clinic of Psychiatry and Psychotherapy, University Hospital OWL of Bielefeld University, Bielefeld, Germany; ^2^Department of Clinical Psychology and Psychotherapy, Bielefeld University, Bielefeld, Germany; ^3^Department of Psychology, The Catholic University of America, Washington, DC, United States

**Keywords:** randomized controlled trial, suicidality, treatment, suicide risk, Collaborative Assessment and Management of Suicidality, collaborative approach

## Abstract

**Background:**

Although use of inpatient crisis hospital intervention for suicide risk is common, the evidence for inpatient treatments that reduce suicidal thoughts and behaviors is remarkably limited. To address this need, this novel feasibility pilot randomized controlled trial compared the use of the Collaborative Assessment and Management of Suicidality (CAMS) to enhanced treatment as usual (E-TAU) within a standard acute inpatient mental health care setting.

**Objectives:**

We hypothesized that CAMS would be more effective than E-TAU in reducing suicidal thoughts and behaviors. As secondary outcomes we also investigated depressive symptoms, general symptom burden, reasons for living, and quality of the therapeutic relationship.

**Methods:**

All patients were admitted due to acute suicidal thoughts or behaviors. They were randomly assigned to CAMS (*n* = 43) or E-TAU (*n* = 45) and assessed at four time points (admission, discharge, 1 month and 5 months after discharge). We used mixed-effects models, effect sizes, and reliable change analyses to compare improvements across and between treatment groups over time.

**Results:**

Intent-to-treat analyses of 88 participants [mean age 32.1, *SD* = 13.5; *n* = 47 (53%) females] showed that both groups improved over time across all outcome measures with no significant between-group differences in terms of change in suicidal ideation, depression, reasons for living, and distress. However, CAMS showed larger effect sizes across all measures; for treatment completers CAMS patients showed significant improvement in suicidal ideation (*p* = 0.01) in comparison to control patients. CAMS patients rated the therapeutic relationship significantly better (*p* = 0.02) than E-TAU patients and were less likely to attempt suicide within 4 weeks after discharge (*p* = 0.05).

**Conclusions:**

CAMS and E-TAU were both effective in reducing suicidal thoughts and symptom distress. Within this feasibility RCT the pattern of results was generally supportive of CAMS suggesting that inpatient use of CAMS is both feasible and promising. However, our preliminary results need further replication within well-powered multi-site randomized controlled trials.

**Trial registration:**

DRKS-ID/ICTRP-ID: DRKS00013727. The trial was retrospectively registered in the German Clinical Trials Register, registration code/ DRKS-ID: DRKS00013727 on 12.01.2018 and also in the International Clinical Trials Registry Platform of the World Health Organization (identical registration code).

## 1. Introduction

Worldwide ~700.000 people each year die by suicide (1), in Germany about 9.300, about three times as high as the number of traffic deaths (2, 3). Experts estimate that statistically for every suicide there are 10–20 suicide attempts (4). The high numbers of deaths by suicide are a major global public health problem, and mental health providers play an important role in detecting and reducing suicide risk (4–6). As discussed elsewhere (7, 8), the immense number of people who suffer with serious thoughts of suicide exceeds the number of those who die by this common cause of death around the world.

Regarding effective treatments for suicide risk, there is still a lack of proven effective treatments and although there has been a near-exponential increase in the number of RCTs in this area in the last decades (9, 10). However, remarkably few clinical trial studies focus on specifically acute inpatient care (11) despite its widespread use around the world. Although inpatient care is effective in decreasing symptom load (12, 13), the weeks following discharge are remarkably high-risk periods for suicide deaths (14–16). A review on treatment for suicide risk (17) listed only three studies examining inpatient treatments (18–20). Of these studies only one took place in an inpatient setting (18); as the others were done in a partial inpatient setting (19), and with recruitment in inpatient setting, but for outpatient treatment (20). This suggests that we have a serious research gap for treating high suicide risk of patients with severe psychopathology that require inpatient care (21, 22).

It is well-known that psychotherapy in general is effective in reducing suicidal thoughts and behavior (17, 23). Traditional treatment concepts consider suicidality as a symptom of a mental disorder assuming that it will be reduced by treating the underlying mental disorder (11). Therapies that target the underlying mental disorder and were shown to be effective to reduce suicidal thoughts and behaviors include Dialectical Behavior Therapy for patients with borderline personality disorder or Mindfulness Based Cognitive Therapy for chronically depressed patients (24–26). However, due to time constraints as well as economic and personnel limitations these treatments are rarely accessible within inpatient care at once, and they are only effective after a lengthy treatment duration. Considering the ubiquity of suicidal risk, however, effective shorter-term interventions are needed. Clinical suicidologists have therefore repeatedly questioned whether treatment focusing on mental disorders is most efficient in reducing the risk of suicide in situations with limited time and acute risk (27–29). These authors argue for treating suicide risk trans-diagnostically as an independent syndrome, focusing on the causes of suicidal thoughts and behaviors irrespective of the underlying disorder. Consequently, psychotherapeutic interventions, that directly target suicidal thoughts and behaviors have been found to be more effective in preventing suicide attempts and suicides than those that address these factors indirectly (28).

There are now a number of suicide-focused treatments that have been studied in randomized controlled trials (RCTs). For example, there is the Attempted Short Intervention Program (ASSIP) with three to four outpatient treatment sessions that significantly decreases suicide attempts and 2-year follow up (30, 31). There are also two suicide-focused approaches based on Cognitive Behavior Therapy: Cognitive Therapy for Suicidal Patients (CT-SP) (32) and Brief Cognitive Behavior Therapy Suicide Prevention (BCBT-SP) (33, 34). In addition, a first pilot study showed promising results on Post Admission Cognitive Therapy (PACT) for suicidal patients in the inpatient setting (35). A recent review of common factors across suicide-focused care supported by RCTs has been published in this journal (36).

Another suicide-focused clinical intervention featured in the present RCT is the *Collaborative Assessment and Management of Suicidality (CAMS)*. CAMS focuses on treating the difficulties and challenges that lead to suicidality [referred to as “drivers” within the language of CAMS (27)]. CAMS is a semi-structured therapeutic framework in which the therapist and the patient jointly engage in a collaborative process to reduce suicidal risk by treating drivers and enhancing the patient's motivation to live. To date, CAMS is supported by a range of clinical trials including ten non-randomized studies and six randomized controlled trials (37–52) as well as two meta-analyses (53, 54). Results suggest that CAMS leads to a rapid and sustained reduction of suicidal ideation, overall symptom distress, and related risk factors such as depression and hopelessness within four to eight sessions. It significantly reduces overall symptom distress even twelve months after treatment (47, 51). A recently published meta-analysis found significant small to medium effect sizes for CAMS in relation to suicidal ideation of d = 0.25, medium effect sizes for general symptom distress (d = 0.29), treatment acceptance (d = 0.42) and a strong effect on increasing hope/reducing hopelessness with d = 0.88 (54). The impact of CAMS on self-harm and suicide attempts is promising but so far limited—e.g., with studies being underpowered (48). There is recent evidence that CAMS can treat suicide risk in a cost-effective manner (55). Initially, CAMS was developed for use in outpatient settings, but the feasibility and usefulness of CAMS in longer inpatient settings have already been demonstrated (43–45). Ryberg et al. (51) showed CAMS to be effective in a context with a broad standard psychiatric sample among a mixed sample with both inpatients and outpatients.

This present study reports results of a feasibility pilot randomized controlled trial that investigated the feasibility and efficacy of CAMS for treatment of inpatients who were acutely suicidal within an inpatient crisis intervention setting. We hypothesized that CAMS would be more effective than E-TAU in reducing suicidal thoughts and behaviors. Secondary hypotheses were that CAMS would reduce general symptom distress, depression, increase reasons for living, and improve the therapeutic relationship more than E-TAU. The primary aim was to investigate whether CAMS reduces suicidal ideation and behavior more effective than an enhanced Treatment as Usual (E-TAU) among inpatients.

## 2. Material and method

### 2.1. Study site and design

The study was approved by the University of Muenster Ethics Committee and is in accordance with the Declaration of Helsinki. This RCT was conducted on a crisis intervention ward at the Clinic of Psychiatry and Psychotherapy, Bielefeld University, Germany. We compared two brief psychotherapeutic interventions for patients with suicidal thoughts and behaviors: CAMS and E-TAU, both integrated in an inpatient care. A total of 88 eligible participants were randomly assigned in a 1:1 ratio. The period of enrolment and follow-up ran from February 2017 to June 2019. All patients provided written informed consent for the study procedures. The design and rationale of the trial are described in more detail in the published study protocol (56). Primarily we report results from the intent-to-treat analyses (ITT) and additionally some results from the treatment completers (TC) where it makes sense.

### 2.2. Participants and procedures

Patients were referred by their attended psychiatrists in private practices or outpatient services or by hospitals' intensive care units due to acute suicidal thoughts or after suicide attempts. Within the admission interview for inpatient crisis treatment on the ward, patients were consecutively screened for inclusion criteria. In case of a positive screening, patients were invited to participate.

Women and men aged 18–65 years speaking fluent German were included—independent of their specific mental disorders. The duration of the treatment had to be at least 10 days in order to ensure that patients received treatment to a sufficient extent (between four and nine sessions of either CAMS or E-TAU following the admission interview). We excluded patients, who were chronically suicidal, because we focused our study on patients in an acute suicidal crisis and CAMS previously has been found to be less effective for chronic risk (52). Chronical suicidality was defined as follows: (i) inpatient treatment >12 weeks within the last 12 months or (ii) > six admissions during this period or (iii) living in a residential supported housing setting. In addition, patients with acute psychotic symptoms during the last 12 months, eating disorders with BMI <16 and/or a current substance dependence were excluded as well as patients with developmental disabilities, intelligence disorders, dementia or organic disorders. We also excluded patients who underwent further long-term residential or day clinic treatments, patients who were admitted against their own's will according to the law of aid and protection for mental illness (PsychKG North Rhine-Westphalia) or according to the federal care law.

### 2.3. Assessment and measures

With a summary of all available information, the inclusion and exclusion criteria and the current psychiatric diagnoses were made by at least one psychiatrist and one psychologist in the first admission interview. Randomization was performed by an independent researcher by throwing the dice immediately after the patients have agreed to participate in the study and returned the completed questionnaires. After randomization, the study participants were followed for about 6 months at four time points (admission, discharge, 1 month, and 5 months after discharge).

#### 2.3.1. Diagnostic procedures

The Structured Clinical Interviews for DSM-IV Axis I Disorders (SCID-I) and Axis II Disorders (SCID-II) were administered by an independent trained research assistant (psychologist, B.Sc.) to diagnose Axis I and Axis II disorders [German versions: (57–59)]. To assure sufficient intelligence the Mehrfach-Wortschatz-Intelligenztest (MWT-B) (60) was administered. Diagnostics were performed at baseline within the 1st days after admission.

#### 2.3.2. Questionnaires

Participants completed a set of questionnaires (i) (pre) on the admission day or the following day, (ii) 10 to 40 days later at the day before discharge (post), (iii) 4 weeks after discharge (FU1), and (iv) 5 months after the end of their treatment (FU2 by mail).

The primary outcome in the trial was the change of suicidal ideation severity and the occurrence of suicidal behaviors from baseline measure (pre) up to FU2. Suicidal ideation was measured at each assessment by the *German Version of the Beck Scale for Suicide Ideation* (BSS) (61), a patient-rated questionnaire with 21 items that measures a patient's suicidal ideation at its worst point during the past 2 weeks (Likert scale from 0 to 2, total score of maximum 38). To capture suicidal behaviors, we documented self-reported suicidal behaviors during the course of treatment and follow-up periods. Additionally, a reliable change index (RCI) was analyzed for treatment completers only: In absence of a valid cut-off score for clinically significant change or treatment response of the BSS, we a-priori defined a reliable change (RCI) in suicidal ideation as a change of ± 18.80 points of the BSS Scale (calculated with a test-retest reliability of the BSS = 0.54 (62, 63).

Secondary outcomes were scores on standard self-report measures of psychopathology (German versions): Beck Depression Inventory (BDI-II) (64, 65) to capture depressive symptoms, Mini Symptom Checklist (Mini-SCL) (66) to capture general symptom burden, Brief Reasons for Living Inventory (B-RFL) (67) to capture the amount of reasons for living, and Scale to Assess the Therapeutic Relationship in Community Mental Health Care, Patient-Version (D-STAR-P) (68, 69) to capture the quality of the therapeutic alliance.

### 2.4. Interventions

#### 2.4.1. Collaborative Assessment and Management of Suicidality

CAMS is designed to enable the patient to constructively deal with their suicidal urges within a non-adversarial and collaborative therapeutic dynamic. Patients are actively engaged as “co-authors” of their own suicide-focused treatment plan. Within CAMS, the patient and therapist sit next to each other for assessment and treatment planning as they work together with treatment focused on patient-identified “drivers.” CAMS relies on a multi-purpose assessment, treatment-planning and tracking tool, called the Suicide Status Form (SSF)—the clinical “roadmap” that guides the intervention. With two 30–60-min sessions weekly, each is initiated by the patient rating the SSF “Core Assessment” on 1–5 rating scales for psychological pain, stress, agitation, hopelessness, self-hate, and their overall judgment of their risk for suicide. In the first session, the patient was prompted to also provide a qualitative written descriptions for each suicidal marker (e.g., what causes their pain, stress, etc.). Additionally, patients are asked to rank these items on the SSF from the most to least important. The first session in CAMS is continued with an assessment of the extent to which one's suicidality depends on thoughts or feelings toward oneself or others, as well as a collection of “reasons for living” and “reasons for dying.” In addition, the strength of the patient's “wish to live” and “wish to die” is assessed and the therapist and patient also jointly assessed other factors known to increase suicide risk, such as whether there were current suicide plans, lethal means available, or substance use or if the patient is socially isolated.

A problem-focused treatment plan addressing what makes the patient suicidal is jointly developed at the end of the first session (and routinely re-considered at the end of each interim session of CAMS). Furthermore, as part of CAMS-guided treatment planning, the CAMS Stabilization Plan is developed (70) and further crafted over treatment to increase the patient's coping skills as ongoing treatment centers on the patient-defined drivers of suicide.

The duration of CAMS-guided treatment depends on the treatment progress and varies; CAMS is ended when the clinician and the patient agree that the overall risk of suicide is diminished and the patient is able to reliably manage their suicidal thoughts, feelings, and remain behaviorally stable (i.e., acute danger of a suicidal risk is reduced, and adaptive coping skills have been developed). This is achieved when the patient score below 3 on subjective suicidal risk rating (on a 5-point scale) and the patient is able to manage suicidal thoughts or feelings while remaining behaviorally stable. When this outcome was reliably achieved, discharge from the inpatient setting was set in motion.

#### 2.4.2. Enhanced treatment as usual

Patients in the E-TAU condition also received supportive, cognitive-behavior-therapy (CBT) based sessions similar to the number of CAMS sessions (four to nine 30–60-min lasting sessions during treatment). There was no pre-defined manual for the E-TAU treatment. In addition to establishing a viable therapeutic relationship and acute relief, diagnostic, psychoeducation and initial therapeutic steps, development of a treatment perspective for the underlying disease or general life problem were focused. Depending on the current risk situation, the aim was to promote the patient's safety, to encourage the patient to reflect and to build up confidence and motivation for treatment and the effecting of changes. Depending on the problem areas described by the patient, the practitioners independently determined the focal points and contents of the therapeutic sessions together with the patients. The therapists were free to choose methods and strategies to promote self-control and the use of social support as well as to learn strategies for emotional stabilization.

#### 2.4.3. Standard inpatient care

All patients received a combination of Standard Inpatient Care (SIC) in addition to either CAMS or E-TAU. SIC included unspecific therapy elements, such as occasionally supportive consultations with the nursing staff and a daily individual therapy plan designed on the patient's stability and wishes so that the patient could participate in additional offers such as body and movement therapy, Jacobson relaxation, music therapy, occupational therapy and offers from clinical social work and pastoral care. In addition, weekly visits by a senior physician took place. The interventions were embedded in the context of a therapeutic environment that offered continuous care and plenty of opportunities for spontaneous contact with fellow patients. In both groups, patients received medication according to their diagnosis and current symptoms, including benzodiazepines and on-demand medication, if necessary.

### 2.5. Choice of comparator and treatment dose

The aim of this trial was to test the efficacy of CAMS for suicidal inpatients in a crisis intervention unit. For this purpose, it was necessary to compare CAMS as provided on the ward with the treatment that would be available without this module, i.e., a TAU condition. To increase experimental internal validity, TAU in this study was “enhanced” (i.e., E-TAU) to ensure comparability of treatment dose. Patients in the E-TAU condition received as many treatment sessions as patients in the CAMS condition, ~two per week. Thus, E-TAU has been designed to balance and minimize threats to both the internal and external validity of the study.

### 2.6. Therapists

The therapists were a licensed psychotherapist and a psychologist (M.Sc.). Both therapists offered CAMS and TAU treatment and so they served as their own controls which showed fidelity and increased the internal validity. Therapists treated almost the same number of patients (41 vs. 45 patients; two patients were treated by both therapists due to the absence of the other therapist) and were equally distributed across treatment conditions (χ^2^(2) = 0.01, *p* = 0.99). The assignment to the therapist was consecutive, i.e., the clinician who conducted the admission interview also was responsible for further treatment. There were no significant differences of dropout rates between therapists (χ^2^(2) = 5.20, *p* = 0.07).

Both therapists had been trained in CAMS using the E-learning training by David Jobes which both therapists completed before the start of the study. Additionally, both had worked intensively with the CAMS manual and previously practiced the application of the Suicide Status Form (SSF) with at least three patients. Each therapy session was audiotaped. Every fourth treatment session of the therapists in both treatment conditions was supervised and rated for adherence according to the CAMS Rating Scale (CRS) (71, 72) by external evaluators. The CRS is an observer rating scale and consists of three parts and 14 items in total. Part I covers the treatment philosophy, part 2 the clinical/ session framework and part 3 the overall rating. The items were rated on a 6-point scale from 0 = poor to 6 = excellent. Supervisors had access to all documents (e.g., assessments, audiotapes of therapy sessions, therapy session sheets) to check diagnostic accuracy, consistency of data entry, and adherence to treatment. Adherence to CAMS was given throughout the course of the study for both therapists and the supervisors also ensured with help of the CRS that the therapists did not use any CAMS strategies in the E-TAU sessions establishing experimental fidelity between the treatment arms.

### 2.7. Statistical analysis

Alpha was set at 0.05 to preserve power. We determined that with a sample size of 50 (=2 × 25) clients, the outcome analyses had at least 80% power to detect an effect size of 0.8. For compensating participants who withdraw their consent, discontinue treatment, or have to be excluded due to longer inpatient treatment or the fulfillment of exclusion criteria, we planned 36 subjects per group.

Baseline characteristics of the groups were compared to examine any pre-treatment differences despite randomization. Continuous data were compared across treatment groups using t-tests or Mann-Whitney-U-tests, depending on fulfillment of assumptions. Dichotomous data were compared across treatment groups using χ^2^-tests and for cell occupancies smaller than 5 with Fisher's exact test.

All treatment-related analyses were conducted on the treatment completer sample (TC) and the intention-to-treat sample (ITT). To avoid a bias, we included all participants in our ITT analyses who were randomized and completed baseline assessment, even if they dropped out immediately after randomization and were unavailable for one or more of the follow-up interviews. We applied mixed-effects models that allowed the inclusion of all available data. The models predicted treatment response using group as a fixed factor, time points as a within-participants repeated factor and participants as random factor with random intercepts and slopes for each participant. Mixed-effects models have several advantages: All available data are incorporated in the analyses, and they account for serial correlation within participants, are relatively robust to randomly missing data, and can incorporate certain non-random missing data without biasing model estimates. Thus, we did not impute missing data as multiple imputations do not offer advantages over linear mixed models (73–75). We calculated mixed-effects models for the primary outcome of the BSS (suicidal ideation), including participants as random effects and treatment, time and treatment x time as fixed effects, whereby each participant was nested in treatment. The secondary outcomes of depressive symptoms, the general symptom distress and reasons for living were analyzed in the same way.

For descriptive analyses of mean change differences between-groups independent sample *t*-tests were calculated on a treatment-completer basis. Clinical significance was estimated by calculating within- and between-treatment effect-sizes (Cohen *d*). Within-treatment effect sizes were calculated by dividing the difference between the means of pre-treatment and post-treatment scores (or follow-up-treatment scores) by the pooled standard deviation of means. Between-treatment effect sizes were corrected for pre-test differences between the groups [*d*_korr_ sensu Klauer (76)]. In absence of a valid cut-off score for clinically significant change or treatment response of the BSS, the number of subjects with clinically significant improvement as well as worsening based on the reliable change index (RCI) were compared between groups using χ^2^–tests or Fisher's exact tests. For this purpose, the RCI was calculated based on the pre-treatment scores of the study sample. We used two-tailed tests for statistical significance with alpha set at *p* < 0.05. All calculations were performed using SPSS 20.0 (77).

## 3. Results

### 3.1. Patient flow and participant characteristics

**Figure 1:**
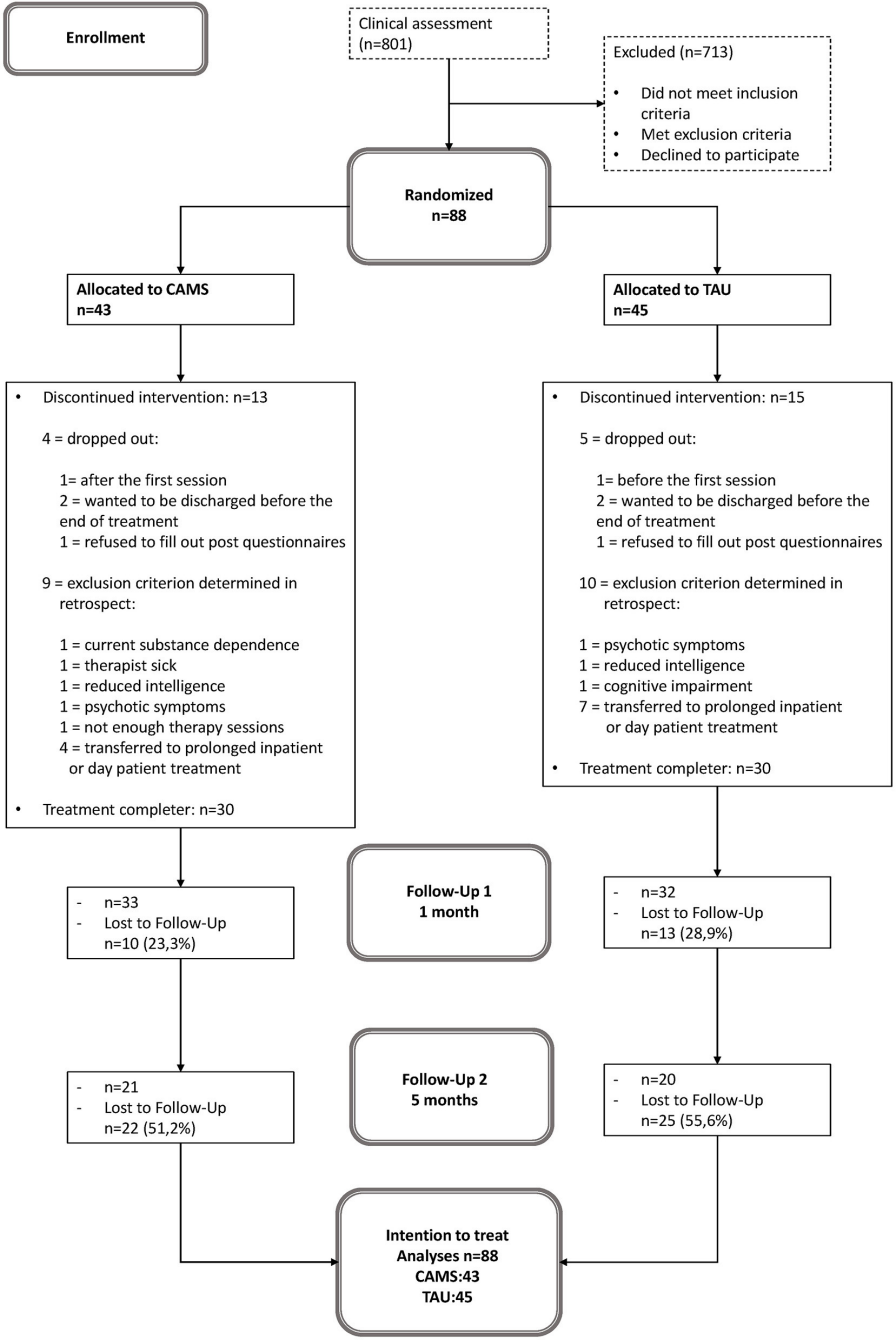
Patient flow. CAMS, Collaborative Assessment and Management of Suicidality; E-TAU, Enhanced-Treatment as Usual.

Of 801 patients who were referred for residential crisis treatment within the recruitment period (February 2017 to January 2019), 88 patients were found to be eligible for participation in the study and were randomly assigned to either CAMS (*n* = 43) or E-TAU (*n* = 45). There were four individuals randomized to CAMS and five individuals randomized to E-TAU dropped out of treatment; nine individuals were randomized to CAMS and 10 individuals were randomized to E-TAU discontinued participation in the study because of exclusion criterion determined in retrospect (see Figure 1). Because the exclusion criteria for these patients became apparent only during the course of treatment, they were all included in the ITT analyses.

Demographic and clinical characteristics of patients at baseline are shown in Tables 1, 2. 53% were women, the mean age was 32 years. Most participants fulfilled the criteria for a depressive disorder (73%) and considerably more than one half those of a personality disorder (60%) with most common borderline personality disorder (35%). Almost half of the patients qualified for at least one secondary diagnosis (49%). Prior suicide attempts were reported by almost two thirds (65%), one third (34 %) had a history of multiple (>2) suicide attempts. Nearly one third (33%) had been hospitalized in psychiatric care at least once. Most of the patients used psychotropic medications, most frequently antidepressants (75%) and antipsychotics or mood stabilizers (30%). On average, participants at baseline had a mean BSS score of 18.73 (*SD* = 10.46) and a mean BDI score of 36.17 (*SD* = 12.02). At baseline CAMS group and E-TAU group did only differ with regard to intelligence screening (MWTB; *t (*74) = −2.32, *p* = 0.023), and the education level with higher levels in the CAMS group. Thus, both were included as fixed effects in the mixed-effects models.

**Table 1:** Demographic characteristics for participants at baseline—Intention-to-treat (*n* = 88).

**Demographic characteristics**	**CAMS (*n =* 43)**	**E-TAU (*n =* 45)**	**CAMS vs. E-TAU Statistics**
Sex, female	20 (46.5)	27 (60%)	χ^2^ (1) = 1.608, *p =* 0.205
Age (years)	29.14 (11.35)	34.84 (14.85)	Z = −1.760^b^, *p =* 0.078
Intelligence, MWT-B^a^	25.05 (4.74)	22.34 (5.42)	*t (74) =*−2.32, *p =* 0.023^*^
Single	26 (60.5)	21 (46.7)	χ^2^ (1) = 1.682 *p =* 0.195
Married or partnership	15 (34.9)	16 (35.6)	χ^2^ (1) = 0.004 *p =* 0.947
Separated, divorced, or widowed	2 (4.6)	8 (17.8)	χ^2^ (1) = 3.762, *p =* 0.052
School education in years	11.37 (1.77)	10.38 (1.63)	Z = −2.928^b^, *p =* 0.003^**^

Data are expressed as mean (SD) or as number (%). CAMS, Collaborative Assessment and Management of Suicidality; E-TAU, Enhanced-Treatment as Usual; MWT-B, Mehrfach-Wortschatz-Intelligenztest.

a Number of Items and maximum Score of 37 with higher scores reflecting higher intelligence.

b Non-parametric Mann-Whitney U-test.

* *p* ≤ 0.05.

** *p* ≤ 0.01.

**Table 2:** Clinical characteristics for participants at baseline—Intention-to-treat (*n* = 88).

**Clinical characteristics**	**CAMS (*n* = 43)**	**E-TAU (*n* = 45)**	**CAMS vs. E-TAU Statistics**
**Diagnosis (ICD-10)**			
Depressive disorder (F32-F33)	28 (65.1)	36 (80.0)	χ^2^ (1) = 2.456, *p =* 0.117
Borderline-personality disorder (F60.31)	15 (34.9)	16 (35.6)	χ^2^ (1) = 0.004, *p =* 0.947
Another axis-II disorder (F6)	14 (32.6)	8 (17.8)	χ^2^ (1) = 2.562, *p =* 0.109
Bipolar disorder (F31)	1 (2.3)	1 (2.2)	χ^2^ (1) = 0.001, *p =* 0.974
Posttraumatic stress disorder (F43.1)	4 (9.3)	3 (6.7)	χ^2^ (1) ^= 0.2^09, *p =* 0.648
Psychotic disorder (F20)	1 (2.3)	1 (2.2)	χ^2^ (10) ^= 0.0^01, *p =* 0.974
At least one secondary diagnosis	23 (53.5)	20 (44.3)	χ^2^ (1) ^= 0.4^19, *p =* 0.518
Three or more diagnoses	6 (14.0)	6 (13.3)	χ^2^ (1) = 0.007, *p =* 0.932
**Medication**			
Antidepressants	29 (67.4)	34 (75.6)	χ^2^ (1) = 0.712, *p =* 0.399
Antipsychotics/mood stabilizer	11 (25.6)	16 (35.6)	χ^2^ (1) = 1.029, *p =* 0.311
Benzodiazepines	2 (4.7)	3 (6.7)	χ^2^ (1) = 0.167, *p =* 0.683
**Previous suicide attempts**			
No suicide attempt	18 (41.9)	13 (28.9)	χ^2^ (2) = 4.428, *p =* 0.109
One suicide attempt	12 (27.9)	15 (33.3)	
Two or more suicide attempts	13 (30.2)	17 (37.8)	
**Number of previous inpatient treatments**			
Not any	29 (67.4)	20 (44.4)	χ^2^ (2) = 5.182, *p =* 0.075
One	4 (9.3)	10 (22.2)	
>Two	10 (23.3)	15 (33.3)	
**Primary and secondary outcomes**			
Suicidal Ideation, BSS^a^	18.86 (10.55)	18.60 (10.19)	*t (86) = −0.116, p =* 0.908
Depression, BDI-II^b^	36.00 (11.33)	36.33 (12.77)	*t (86) = 0.129, p =* 0.897
General symptom distress, Mini-SCL^c^	32.30 (12.17)	37.04 (13.02)	*t (86) = 1.76, p =* 0.081
Reasons for Living, Brief RFL^d^	24.00 (6.94)	25.36 (8.39)	*t (86) = 0.824, p =* 0.412

Data are expressed as mean (*SD*) or as number (%). CAMS, Collaborative Assessment and Management of Suicidality; E-TAU, Enhanced-Treatment as Usual; BSS, Beck Scale for Suicide Ideation (61); BDI-II, Beck Depression Inventory (64); Mini-SCL (66); B-RFL, Brief Reasons for Living Inventory (67).

a Maximum score = 38, higher scores indicate greater suicidal ideation.

b Maximum score = 63, higher scores indicate higher levels of depression.

c Maximum score = 72, higher scores indicate higher symptom distress.

d Maximum score for Reasons for Living = 48, Minimum Score for Reasons of Living = 12, higher scores indicate more or more important reasons to live.

### 3.2. Attrition rates/dropout rates and scope of treatment

The drop-out rates for the CAMS and E-TAU group (see Figure 1) were 19% for CAMS and 22% for E-TAU at post/Discharge; 23% for CAMS and 29% for E-TAU at 1 month, and 51% for CAMS) and 56% for E-TAU at 5 months. The number of missed assessments (questionnaires) (FU1: χ^2^(1) = 0.59, *p* = 0.44; FU2: χ^2^(1) = 1.59, *p* = 0.21) and the reasons for treatment dropout did not differ between treatment conditions (χ^2^(3) =1.39, *p* = 0.71). Treatment duration varied between ten and 40 days (*M* = 22.60, *SD* = 7.35) with no difference between groups (*t*(58) = −0.63, *p* = 0.53). Participants received between four and nine sessions (*M* = 5.37, *SD* = 1.51) without any statistical difference between groups (*t*(53) = −1.20, *p* = 0.23).

### 3.3. Clinical outcomes

**Figure 2:**
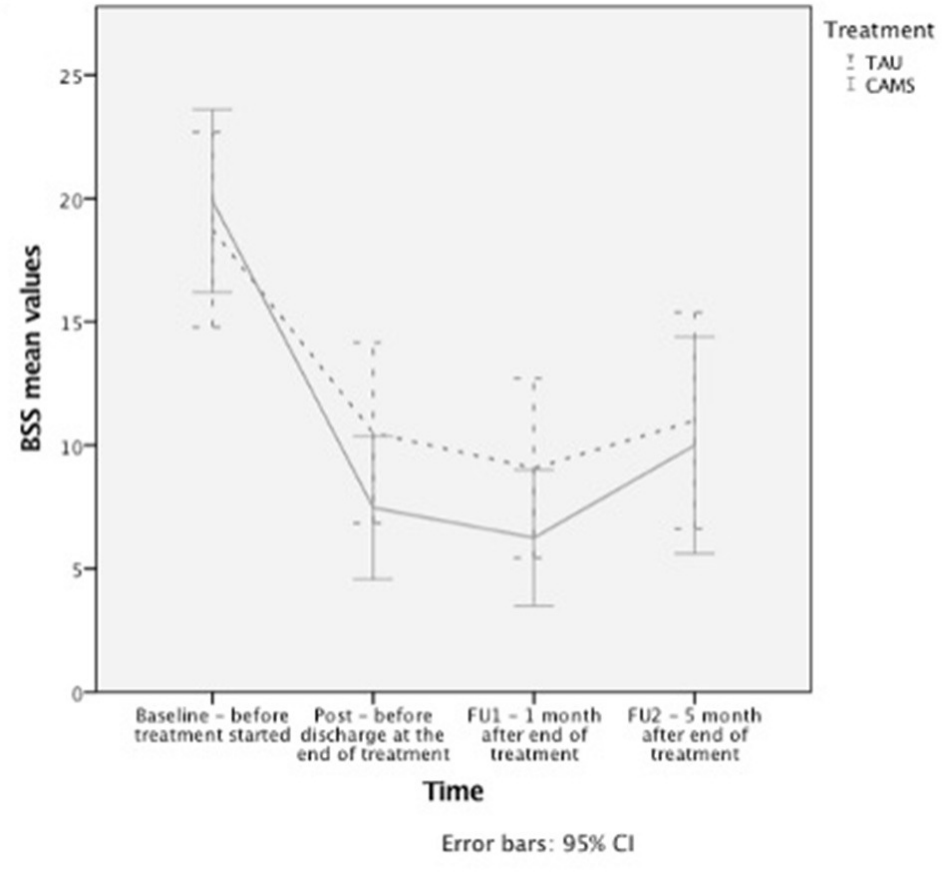
Longitudinal changes in suicidal ideation. Changes in suicidal ideation as measured with the BSS over time, presented with error bars for 95% confidence interval.

All analyses were conducted for ITT and TC samples separately. The results for the TC sample are only shown for reliable change and mean change differences, all other ones in the Appendix (available on demand). Descriptive data and within-group Cohens *d* effect sizes as well as the results of the mixed-effects models for primary and secondary outcomes are presented in Table 3. Pre-treatment differences of intelligence (MWTB) and education (education in years) affected the results of the mixed-effects models, and the inclusion of both factors as fixed factors lead to significant main and interaction effects, especially the main and interaction effects of intelligence (MWTB) were all highly significant in each model (*p* < 0.001). Table 4 shows the mean change difference scores and between treatment effect sizes for primary and secondary outcomes for the TC sample. All treatment effects in both treatment groups appear to be strongest at 1 month follow up and with some rebound between FU1 (1 month) and FU2 (5 months) in both groups (see Figure 2).

**Table 3:** Primary and secondary outcome data at all time points and within-group effect sizes and results of mixed-effects models for primary and secondary outcomes—Intention-to-treat sample (*n* = 88).

**Measurement**	**CAMS (*n* = 43)**	**E-TAU (*n* = 45)**	**Statistics pre-FU2**		
**Primary outcome**			**Main effects**		**Interaction**
**BSS, suicidal ideation**			**Treatment**	**Time**	**Treatment x time**
Pre-treatment	18.86 (10.85)	18.6 (10.19)	}1.38_1, 84_	36.41${}_{3,172}^{{}^{***}}$	0.343_3, 172_
Post-treatment (discharge)	8.26 (8.88)	11.23 (9.90)			
FU1 (4 weeks follow-up)	6.53 (7.45)	8.63 (9.23)			
FU2 (20 weeks follow-up)	10.00 (9.65)	9.90 (9.00)			
Cohens *d (*within-groups, pre-post)	1.1	0.7			
Cohens *d (*within-groups, pre-FU1)	1.3	1.0			
Cohens *d (*within-groups, pre-FU2)	0.9	0.9			
**Secondary outcomes**			**Main effects**		**Interaction**
**BDI-II, depression**			**Treatment**	**Time**	**Treatment x time**
Pre-treatment	36.00 (11.33)	36.33 (12.78)	}0.35_1, 87_	44.22${}_{3,178}^{{}^{***}}$	1.44_3, 178_
Post treatment (discharge)	22.25 (13.15)	24.06 (15.10)			
FU1 (4 weeks follow-up)	17.36 (13.29)	20.39 (15.15)			
FU 2 (20 weeks follow-up)	20.05 (12.39)	22.60 (14.90)			
Cohens *d (*within-groups, pre-post)	1.1	0.9			
Cohens *d (*within-groups, pre-FU1)	1.5	1.1			
Cohens *d (*within-groups, pre-FU2)	1.4	1.0			
**Mini-SCL, symptom distress**			**Treatment**	**Time**	**Treatment x time**
Pre-treatment	32.30 (12.17)	37.04 (13.02)	}12.36${}_{1,92}^{{}^{***}}$	29.56${}_{3,178}^{{}^{***}}$	0.51_3, 178_
Post-treatment (discharge)	19.94 (13.02)	23.78 (15.00)			
4 weeks (follow-up)	18.73 (13.72)	20.48 (15.85)			
20 weeks (follow-up)	17.33 (12.13)	21.45 (16.23)			
Cohens *d (*within-groups, pre-post)	1.0	1.0			
Cohens *d (*within-groups, pre-FU1)	1.1	1.2			
Cohens *d (*within-groups, pre-FU2)	1.2	1.1			
**BRFL, reasons for living**			**Treatment**	**Time**	**Treatment x time**
Pre-treatment	24.00 (6.94)	25.36 (8.39)	}0.03_1, 74_	2.75${}_{3,165}^{{}^{*}}$	2.59_3, 165_
Post-treatment (discharge)	27.86 (7.69)	25.11 (8.56)			
4 weeks (follow-up)	28.33 (8.87)	25.81 (8.45)			
20 weeks (follow-up)	25.85 (10.11)	24.95 (8.94)			
Cohens *d (*within-groups, pre-post)	0.5	0.0			
Cohens *d (*within-groups, pre-FU1)	0.6	0.1			
Cohens *d (*within-groups, pre-FU2)	0.2	0.0			
**D-STAR-P, therapeutic relationship**					
Post-treatment (discharge)	32.89 (4.10)	29.97 (5.72)
Cohens *d* (between groups)	0.6	

Primary and secondary outcome data are expressed as mean (SD). All results of the mixed models are presented as F-values (F_d1, d2_). Intelligence (MWTB) and School education (in years) were entered as fixed factors in all models.

^*^p ≤ 0.05. ^***^p ≤ 0.001.

Positive Cohens *d* values indicate improvement from pre to post or Follow-up.

CAMS, Collaborative Assessment and Management of Suicidality; E-TAU, Enhanced-Treatment as Usual; BSS, Beck Scale for Suicide Ideation; BDI-II, Beck Depression Inventory; Mini-SCL, Mini-Symptom Checklist; B-RFL, Brief Reasons for Living Inventory.

**Table 4:** Mean change difference scores and between-groups (CAMS vs. E-Tau) effect sizes (Cohen d) of primary and secondary outcome variables (treatment completers only).

**Variable**	
**Suicidal ideation, BSS** ^a^	
Change difference (post—pre), Mean (SE)	4.20 (2.35)
[95% CI]^b^	[−0.52 to 8.92]
Cohens *d*^c^	0.5
Change difference (FU1—pre), Mean (SE)	3.63 (2.63)
[95% CI]^d^	[−1.65 to 8.91]
Cohens *d*	0.5
Change difference (FU2—pre), Mean (SE)	2.67 (3.01)
[95% CI]^e^	[−3.43 to 8.78]
Cohens *d*	0.2
**Depression, BDI-II** ^f^	
Change difference (post—pre), Mean (SE)	0.97 (2.74)
[95% CI]^b^	[−4.53 to 6.46]
Cohens *d*	0.1
Change difference (FU1—pre), Mean (SE)	3.13 (3.33)
[95% CI]^d^	[−3.55 to 9.80]
Cohens *d*	0.6
Change difference (FU2—pre), Mean (SE)	3.08 (3.57)
[95% CI]^e^	[−4.15 to 10.43]
Cohens *d*	0.2
**Symptom distress, mini-SCL** ^g^	
Change difference (post—pre), Mean (SE)	−2.17 (3.18)
[95% CI]^b^	[−8.53 to 4.20]
Cohens *d*	0.2
Change difference (FU1—pre), Mean (SE)	−4.54 (3.40)
[95% CI]^d^	[−11.36 to 2.28]
Cohens *d*	0.3
Change difference (FU2—pre), Mean (SE)	−1.65 (4.28)
[95% CI]^e^	[−10.32 to 7.02]
Cohens *d*	0.1
**Reasons for living, B-RFL** ^h^	
Change difference (post—pre), Mean (SE)	−4.60 (1.87)
[95% CI]^b^	[−8.35 to 0.85]
Cohens *d*	0.6
Change difference (FU1—pre), Mean (SE)	−3.30 (2.17)
[95% CI]^d^	[−7.65 to 1.05]
Cohens *d*	0.4
Change difference (FU2—pre), Mean (SE)	−4.51 (3.17)
[95% CI]^e^	[−10.95 to 1.93]
Cohens *d*	0.3

CAMS, Collaborative Assessment and Management of Suicidality; E-TAU, Enhanced- Treatment as Usual; BSS, Beck Scale for Suicide Ideation; BDI-II, Beck Depression Inventory; Mini-SCL, Mini-Symptom Checklist; B-RFL, Brief Reasons for Living Inventory.

a Maximum Score for Suicidal Ideation of 38 with higher Scores reflecting greater ideation.

b Post—pretreatment, treatment completers only; *n* = 30 for CAMS, *n* = 30 for E-TAU.

c Collaborative Assessment and Management of Suicidality vs. Enhanced-Treatment as Usual. D_korr_ sensu Klauer (76). Positive Cohens *d* values indicate superiority of Collaborative Assessment and Management of Suicidality. Effect sizes were considered large with a *d* of 0.80 or greater, moderate with a *d* of 0.50 to 0.79, and small with a *d* of 0.20 to 0.49.

d 1-month follow-up—pretreatment, treatment completers only; *n* = 29 for CAMS, *n* = 28 for E-TAU.

e 5-month follow-up—pretreatment, treatment completers only; *n* = 21 for CAMS, *n* = 18 for E-TAU.

f Maximum Score for Depression of 63 with higher scores indicating more severe levels of depressive symptoms.

g Maximum Score for General Symptom Distress of 72 with higher scores indicating more Symptom Distress.

h Maximum Score for Reasons for Living of 48, Minimum Score for Reasons of Living of 12, with higher scores indicating more or more important reasons to live.

#### 3.3.1. Suicidal thoughts and behaviors (primary outcome)

The mixed-effects models on suicidal ideation assessed by the BSS showed a significant reduction across time in both TC and the ITT samples for post-treatment and all follow-up time-points (Table 3, Figure 2). However, there was no significant time x treatment interaction indicating that the treatment groups did not differ significantly regarding improvement over time.

Within group effect sizes for changes on the BSS score in both treatment conditions were considered medium to large in E-TAU and large in CAMS (for ITT between 0.7 to 1.0 in E-TAU and between 0.9 to 1.3 in CAMS) with the largest effect found in CAMS (*d* = 1.3 with a 65.4% reduction in the BSS score from pre to FU1, compared with the E-TAU group (*d* = 1.0 46.8% reduction of suicidal ideation).

The TC sample revealed higher ES, e.g., BSS *d* = 1.0 to 1.6 in CAMS and *d* = 0.8 to 0.9 in E-TAU. In both groups, effect sizes decrease over time with the lowest scores in FU2.

Comparing CAMS to E-TAU suicidal ideation (according to the BSS) showed a mean change difference of 4.20 (post–pretreatment) and of 3.63 (FU1—pre) and of 2.67 (FU2—pre) in favor for CAMS. Between-treatment effect sizes for suicidal ideation were d=0.5 (for pre-post and pre-FU1) and *d* = 0.2 (for pre-FU2) favoring CAMS (see Table 4). The descriptive data indicate that suicidal ideation more rapidly decreased in the CAMS group than in E-TAU patients (see Table 3).

Comparisons of individual BSS scores before treatment and at post (1-month follow-up/5-months follow-up) indicated that most participants in both groups showed considerable improvement of suicidal ideation. At post/discharge the rate of patients who show reliable change on BSS score was significant higher in CAMS (*n* = 11; 36.7 %) than in E-TAU (*n* = 2; 6.7 %) (χ^2^(1) = 7.95; *p* = 0.01). At 1-month follow-up ten patients in the CAMS group (35.7%) showed reliable improvement in the BSS score vs. five patients (17.2%) in the TAU group and at 5-months follow-up five patients (23.8%) in the CAMS group showed reliable improvement vs. two patients (11.2%) in the TAU condition (FU1: χ^2^(1) = 2.51; *p* = 0.14; FU2: χ^2^(1) = 1.06; *p* = 0.42). For either treatments, no patient showed reliable worsening at post-treatment or follow-up at 1 or 5 months. Noteworthy, there was a high variance of suicidal ideation at all times of assessment.

Three patients in the TAU group reported a suicide attempt within 4 weeks after discharge compared with no patients in the CAMS group, suggesting that patients in the CAMS group were significantly less likely to make suicide attempts after discharge until follow-up 1 (Fisher's exact test, two-tailed *p* = 0.05). Between 1-month follow-up and 5-months follow-up, there were three more reported suicide attempts in the TAU group vs. one in the CAMS group (Fisher's exact test, two-tailed *p* = 0.21) and one completed suicide in the CAMS group.

#### 3.3.2. Depression and general symptom distress

The pattern of the mixed effects in the mixed linear models found in the primary outcome with significant effect of time on symptom severity, no significant interaction effect of time x treatment was also observed for BDI measures and general symptom distress (see Table 3).

#### 3.3.3. Reasons for living

Mixed-effects models on Reasons for Living assessed with the BRFL showed a significant increase across time in both TC and the ITT sample (*p* ≤ 0.05 in ITT and *p* ≤ 0.01 in TC). The significant time x treatment interaction in the TC sample suggests that CAMS helped patients to discover more reasons for living, i.e., more hope and confidence, than treatment with E-TAU. In addition, a significant effect is seen for treatment in the TC sample. E-TAU had only a weak effect on reasons for living (*d* = 0.0 for pre-post and *d* = 0.1 pre-Follow-up 1), whereas CAMS showed considerable changes in mean values and medium within-group effect sizes (*d* = 0.6 for pre-post and pre-Follow-up 1, see Table 3).

#### 3.3.4. Therapeutic relationship

At the end of treatment and also 4 weeks later, patients in the CAMS group rated the therapeutic relationship significantly better than patients in the E-TAU group (post: *t (*57) = −2.50, *p* = 0.02; FU1: *t (*54) = −2.61, *p* = 0.01). Between-group-effect sizes were medium (d = 0.7) favoring CAMS.

#### 3.3.5. Use of psychotherapy, day-and inpatient treatments

There were numerical but not significant differences in receiving psychotherapy at 1-month follow-up (CAMS *n* = 18 vs. E-TAU *n* = 8; χ^2^(2) = 6.15, *p* = 0.46). At 5-month follow-up there was no difference (*n* = 12 patients in both groups; χ^2^(2) = 0.21, *p* = 0.90). Regarding day-patient or further inpatient treatment or readmissions there were no significant differences between the groups (1-month: *n* = 9 in both groups day-patient or inpatient treatment; χ^2^(2) = 1.16, *p* = 0.56; 5-month: CAMS *n* = 2, E-TAU *n* = 7; χ^2^(2) = 3.68, *p* = 0.16).

## 4. Discussion

This pilot randomized controlled trial was conducted to investigate the feasibility and efficacy of the Collaborative Assessment and Management of Suicidality (CAMS) for treatment of inpatients who were acutely suicidal in an inpatient crisis intervention setting. To our knowledge, this is the first RCT to investigate the effects of CAMS in a time-limited inpatient crisis intervention setting. Participants randomized to CAMS received two therapeutic CAMS sessions per week in addition to Standard Inpatient Care (SIP) and participants randomized to E-TAU received SIP and the same number of cognitive-behavioral therapy sessions without CAMS elements. We hypothesized that CAMS would be more effective than E-TAU in reducing suicidal thoughts and behaviors. As secondary outcomes we also investigated depressive symptoms, general symptom burden, reasons for living and quality of the therapeutic relationship.

The study shows that CAMS appears to be both feasible and effective. The rate of reliable reductions in suicidal thoughts at discharge was higher in CAMS and CAMS patients reported an increase in their reasons for living and better therapeutic relationship when compared to E-TAU patients. Moreover, it seems that patients treated with CAMS were less likely to attempt suicide in the critical four-week period after discharge than patients treated with E-TAU. Patients in both treatments improved over time in outcome measures for suicidal ideation, depression, and symptom distress. Contrary to our hypothesis, CAMS did not lead to a stronger change with regard to suicidal ideation, depression and symptom distress than E-TAU in the mixed models. The pattern of descriptive results (means) and effect sizes across measures within and between groups tended to favor CAMS when compared to E-TAU, but this RCT was insufficiently powered to fully detect significant differences.

Around 37% (*n* = 11) of patients showed reliable improvements in suicidal ideation after CAMS, but only a small number of patients in E-TAU reduced their suicidal thoughts (7%; *n* = 2). No patient got reliably worse with regard to suicidal ideation in the follow-up period. These findings generally speak for the effectiveness of therapeutic interventions provided within inpatient crisis treatment. The higher number of patients who reliably improved by CAMS suggests an additional benefit of using CAMS. Presumably, the power of our study was insufficient, so that any potential superiority of CAMS in terms of reliable change rates was not evident within our mixed model analyses.

However, it is noteworthy that with only 4–9 therapeutic sessions—and an active control group—generated results supporting the use of CAMS within the context of an acute inpatient setting.

Indeed, CAMS patients reported a significant increase in their reasons for living and significantly better therapeutic relationship when compared to E-TAU patients. The latter result is interesting in consideration of the fact that the quality of the therapeutic relationship is a major predictor of positive treatment responses and motivates patients to seek further help (78). Moreover, that therapists were the same in both therapy arms of the RCT shows the potential impact of CAMS on the therapeutic alliance (e.g., clinicians served as their own control).

In the 4 weeks after discharge from inpatient treatment, there were fewer suicide attempts after treatment with CAMS than after treatment with E-TAU. This finding might suggest that CAMS may be more protective against suicidal behavior in the critical post-discharge period, which has repeatedly been shown to be a high-risk period for suicidal behavior (14–16). But given the insufficient power of our study, our preliminary promising finding requires further replication. We must also note that there was one suicide in the CAMS group within the 5-month follow-up period. Given the limited sample size, all between-group comparisons in terms of post-discharge suicidal behaviors must be interpreted with caution because of the potential for false positive findings (79).

It is important to note that our results are consistent with previous RCT studies showing the value of CAMS in different contexts (47–49, 51, 52). Our results can best be compared with an RCT by Ryberg et al. using CAMS in a mixed inpatient and outpatient setting in terms of illness severity and treatment program (51). The effect sizes for suicidal ideation between groups found in our study (BSS: *d* = 0.5) are lower but comparable to those found by Ryberg et al. (51). The reasons for our smaller effects may be due to underpowered sample and that treatment as usual in our study was “enhanced” increasing internal validity but perhaps leading to better outcomes in contrast to actual usual treatment. In addition, our study was conducted exclusively and not only partially in an inpatient setting where inpatients received various other interventions in addition to CAMS and E-TAU, which makes it more difficult to find differential between group effects of single interventions.

We are mindful of possible contamination effects between groups being treated on the same ward. But we do not consider this to be a major confound because as we expressly asked patients not to talk about their group membership and participants in the study received treatments over the course of 2 years such that many participants were not treated in parallel.

There are two non-controlled studies and one controlled study by Ellis et al. (43–45); who first used CAMS in an inpatient setting. Our within-group effects for suicidal ideation (BSS pre-post; *d* = 1.4) are comparable with the within-group effects of Ellis et al. (2012) (43) (BSS pre-post, *d* = 1.4) and those of Ellis et al. (44) (BSS pre-post, *d* = 1.7) and those of Ellis et al. (45) (BSS pre-post: *d* = 1.0). This is noteworthy, because the studies by Ellis took place in a clinic with a selective sample and a markedly longer length of stay (*M* = 58.8 days versus *M* = 22.6 days in our trial) as well as more total therapeutic sessions.

Regarding suicidal behavior, our study is in line with the findings of a previous Danish RCT by Andreasson et al. (48) of patients with borderline traits, in which CAMS was comparably effective as a shortened version of DBT in reducing suicide attempts and self-harm. And our results regarding depression and symptom distress are overall comparable to other studies comparing CAMS and TAU in the treatment of suicidal patients (49, 51, 52).

While we must view our feasibility results with caution, we nevertheless see that CAMS overall appeared to have advantages over E-TAU. This may be due to the collaborative engagement between therapist and patient which validates the patient's experience and sets the stage for suicide-focused treatment centering on the drivers of the patient's suicidality. This particular observation is supported by the data that showed an improved therapeutic relationship related to the use of CAMS. Further research is needed to clarify various potential mediation and mechanism variables of CAMS that may facilitate optimal treatment outcomes.

One characteristic of suicides and suicide attempts is that they are relatively rare —a low base rate phenomena. This implies that effects of treatments on suicidal behaviors can usually be shown only with large sample sizes. It is therefore encouraging to find the potential superiority of CAMS over E-TAU with regard to an apparent reduction of suicide attempts following discharge from inpatient care with a moderate sample size. However, this preliminary finding clearly requires further replication. Collectively with ten open clinical trials, six RCT's and two meta-analyses the evidence supporting CAMS is both replicated and robust. But more inpatient RCTs are needed to further replicate our feasibility findings particularly related to decreasing suicidal behaviors post-discharge. Nevertheless, we believe given the totality of the evidence to date, CAMS should be considered for wider use within the context of inpatient psychiatric care. As we have shown in this RCT its use within acute inpatient settings is both feasible and promising as an evidence-based, relatively easy to learn, cost-effective, suicide-focused approach that patients and clinicians prefer to treatment as usual (47, 80).

### 4.1. Limitations

The results of this feasibility pilot study must be considered in the context of several limitations. First, in the power calculation, we assumed too large an effect size for the superiority of CAMS with respect to suicidal ideation, given the fact that the contrast between conditions in an inpatient setting is quite small. Thus, our study is underpowered and the effects of CAMS on suicidal ideation found in our study were smaller than assumed and too small to definitively demonstrate the superiority of CAMS over E-TAU. Second, the assessment of suicidality was based on self-reporting and could therefore be subject to responder bias. We relied on the accuracy of the information provided by the patients, even though we were able to monitor and record at least repeated inpatient admissions with none of them being due to suicidal behaviors during follow-up. Third, one therapist was part of the research team, and an allegiance effect might be possible. Fourth, we found pre-treatment group differences regarding intelligence and education. In our study, CAMS patients seemed to be more intelligent with a higher level of education. A larger randomized sample would make this less likely to occur. Fifth, the overall level of treatment dropout (32% from pre to post) was a bit higher than in comparable studies. Treatment discontinuations were caused by a relatively large number of transfers to further long-term treatment and subsequent exclusion of patients who fulfilled exclusion criteria that were not identifiable due to the acute situation at the time of admission. We therefore had less data available at follow-up time points, which further reduced the power of the trial. Sixth, the choice of a time point at discharge has been unfavorable considering that discharge has been related to the measure of interest (suicidal ideation). In hindsight, a fixed time point (e.g., 4 weeks after inclusion) would have been more desirable. Seventh, the follow-up period of 5 months was relatively short, and it remains unclear, how suicidal thoughts and symptom distress would have developed in the further course, so studies with longer follow-up periods are needed. Eighth, beyond the virtue of randomization, potential confounding factors have not been thoroughly explored within our study design due to power limitations.

### 4.2. Clinical implications

As the results of our study show, CAMS can facilitate a strong and trusting therapeutic relationship with a patient who is suicidal. The first therapeutic session within a CAMS treatment can require additional time, which may not always be feasible within busy inpatient settings. But the time spent in the first session can be valuable because it ensures a thorough assessment of the current suicide risk and creates optimal conditions for further suicide-focused treatment. It is therefore advisable to consider *which* patients may particularly benefit from the inpatient use of CAMS.

Data from our study suggest that CAMS is certainly useful for inpatients who are admitted as inpatients for the first time in the context of a suicidal crisis. While patients with chronic suicidal ideation and more complex profiles may also benefit, but probably somewhat less (48), Dialectical Behavior Therapy is likely a better treatment for chronically suicidal inpatients. Indeed, as noted by Pistorello et al. (52) the suicide-specific focus of CAMS seems to be particularly beneficial for patients who are experiencing (1) a first suicidal crisis, or (2) acute suicidal ideation, and/or (3) patients *without* or only with few Borderline Personality Disorder symptoms.

We are now at a point where different suicide treatments may be optimally used for different suicidal states with CAMS offering relief to the largest patient population we see—those with serious and acute thoughts of suicide (81).

### 4.3. Conclusions

The data of this pilot randomized controlled trial provide preliminary support for the feasibility and efficacy of CAMS for inpatients who are suicidal. In our study, CAMS effectively reduced suicidal thoughts and overall distress, strengthened the therapeutic relationship, and appeared to have a positive impact on suicide attempt behaviors during the high-risk post-discharge period. Thus, our preliminary RCT results offer promise for an additional benefit of a suicide-focused therapeutic intervention, such as CAMS, within inpatient psychiatric settings. However, results of this underpowered feasibility RCT must be viewed with caution. But given the relatively young science of clinical suicidology, we believe these preliminarily findings are important given clinical demands that inpatient providers face every day. But clearly, well-powered multicenter randomized controlled trials are needed to validate and replicate our results. Considering the magnitude of the problem and the pervasiveness of inpatients who are suicidal the promise of using CAMS needs to be further explored to ensure that we better help decrease suicide-related suffering and to pursue our shared goal of saving more lives from suicide.

## Data availability statement

The raw data supporting the conclusions of this article will be made available by the authors, without undue reservation.

## Ethics statement

The studies involving human participants were reviewed and approved by Ethics Committee of the Medical Association of Westfalen-Lippe and the Westfälische Wilhelms-Universität Münster Gartenstraße 210 – 214 48147 Münster. The patients/participants provided their written informed consent to participate in this study.

## Author contributions

MS was responsible for the study conception and the conduction of the trial as well as the statistical analyses and the writing of the manuscript. FN participated in designing the trial and revised the analyses and the manuscript critically. MB participated in the conception of the trial and was responsible for the supervision of the therapists. CS advised on the statistical analyses. DJ participated in the conception of the trial and critically reviewed the manuscript. MD has been the trial sponsor and participated in the conception of the trial and critically revised the analyses and the manuscript. TB was responsible for the study conception and critically reviewed the statistical analyses and the manuscript. All authors have read and approved the manuscript.
