# Supplementary material for: The Collaborative Assessment and Management of Suicidality compared to enhanced treatment as usual for inpatients who are suicidal: A randomized controlled trial
Source: Front Psychiatry. 2023 Mar 2;14:1038302. doi: 10.3389/fpsyt.2023.1038302 (PMC10017970; doi:10.3389/fpsyt.2023.1038302)
Supplement: Supplementary file 1 [file Table_1.pdf]

Appendix

Supplementary Table 1. Demographic characteristics for participants at baseline – Treatment Completer (n=60)

Demographic characteristics	CAMS (n=30)	E-TAU (n=30)	CAMS vs. E.TAU Statistics
Sex, female	14 (46.7)	17 (56.7)	χ^2 =.601, p=.438
Age (years)	29.20 (11.87)	34.10 (14.53)	Z=-1.525[b], p=.127
Intelligence, MWT-B[a]	24.73 (4.09)	22.9 (5.18)	t =-1.485, p = .143
Single	20 (66.7)	15 (50.0)	χ^2 =1.714, p=.190
Married or partnership	9 (30.0)	10 (33.3)	χ^2 =.077, p=.781
Separated, divorced, or widowed	1 (3.3)	5 (16.7)	χ^2 =2.963, p=.085
School education in years	11.33 (1.47)	10.47 (1.33)	Z=-2.308[b], p=.021*

Note. Data are expressed as mean (SD) or as number (%). Abbreviations: CAMS = Collaborative Assessment and Management of Suicidality; E-TAU = Enhanced-Treatment as Usual; MWT-B = Mehrfach-Wortschatz-Intelligenztest.

[a] Number of Items and maximum Score of 37 with higher scores reflecting higher intelligence.

[b] Non-parametric Mann-Whitney U-test.

*$p \leq .05$.

Supplementary Table *2.* Clinical characteristics for participants at baseline – Treatment Completer (n=60)

Clinical characteristics	**CAMS** (n=30)	**E-TAU** (n=30)	**CAMS vs. E-TAU** Statistics
Diagnosis (ICD-10)			
Depressive Disorder (F32-F33)	21 (70.0)	26 (86.7)	χ^2 =2,455, p=.117
Borderline-Personality Disorder (F60.31)	11 (36.7)	12 (40.0)	χ^2 =.071, p=.791
Another Axis-II Disorder (F6)	9 (30.0)	8 (40.0)	χ^2 =.082, p=.774
Bipolar Disorder (F31)	-	1 (3.3)	χ^2 =1.017, p=.313
Posttraumatic Stress Disorder (F43.1)	3 (10.0)	3 (10.0)	χ^2 =.000, p=1.00
Psychotic Disorder (F20)	-	-	-
At least one secondary diagnosis	16 (53.3)	20 (66.7))	χ^2 =1.111 p=.292
Three or more diagnoses	4 (13.3)	6 (20.0)	χ^2 =.480, p=.488
Medication			
Antidepressants	18 (60.0)	21 (70.0)	χ^2 =.659, p=.417
Antipsychotics/ Mood Stabilizer	6 (20.0)	13 (43.3)	χ^2 =.3.774, p=.052
Benzodiazepines	-	-	-
Previous suicide attempts			
No suicide Attempt	15 (50.0)	8 (26.7)	
One suicide attempt	5 (16.7)	11 (36.7)	χ^2 =1.629, p=.443
Two or more suicide attempts	10 (33.3)	11 (36.7)	
Number of previous inpatient treatments			
Not any	22 (73.3)	11 (36.7)	
One	2 (6.7)	6 (20.0)	χ^2 =8.246, p=.016*
$\geq$ Two	6 (20.0)	13 (43.3.)	
Primary and Secondary Outcomes			

Suicidal Ideation, BSS[a]	19.90 (9.89)	18.73 (10.57)	p=.660
Depression, BDI-II[b]	35.43 (10.99)	36.47 (12.72)	p=.738
General symptom distress, Mini-SCL[c]	32.20 (10.39)	37.87 (14.07)	p=.083
Reasons for Living, Brief RFL[d]	23.73 (7.33)	24.63 (8.32)	p=.658

Note. Data are expressed as mean (SD) or as number (%). Abbreviations: CAMS = Collaborative Assessment and Management of Suicidality; E-TAU = Enhanced-Treatment as Usual; BSS = Beck Scale for Suicide Ideation (Beck & Steer, 1991); BDI-II = Beck Depression Inventory (Beck, Steer & Brown, 1996); Mini-SCL (Franke, 2016); B-RFL = Brief Reasons for Living Inventory (Ivanoff et al., 1994).

[a] Maximum score = 38, higher scores indicate greater suicidal ideation.

[b] Maximum score = 63, higher scores indicate higher levels of depression

[c] Maximum score = 72, higher scores indicate higher symptom distress.

[d] Maximum score for Reasons for Living = 48, Minimum Score for Reasons of Living = 12, higher scores indicate more or more important reasons to live

*$p \leq .05$.
